# Disrupted topological organization of structural brain networks in childhood absence epilepsy

**DOI:** 10.1038/s41598-017-10778-0

**Published:** 2017-09-20

**Authors:** Wenchao Qiu, Chuanyong Yu, Yuan Gao, Ailiang Miao, Lu Tang, Shuyang Huang, Wenwen Jiang, Jintao Sun, Jing Xiang, Xiaoshan Wang

**Affiliations:** 1 Department of Neurology, The Affiliated Huaian Hospital of Xuzhou Medical University, Huaian, China; 2 Department of Neurology, Affiliated Nanjing Brain Hospital, Nanjing Medical University, Nanjing, China; 3 Division of Neurology, Cincinnati Children’s Hospital Medical Center, Center, USA

## Abstract

Childhood absence epilepsy (CAE) is the most common paediatric epilepsy syndrome and is characterized by frequent and transient impairment of consciousness. In this study, we explored structural brain network alterations in CAE and their association with clinical characteristics. A whole-brain structural network was constructed for each participant based on diffusion-weighted MRI and probabilistic tractography. The topological metrics were then evaluated. For the first time, we uncovered modular topology in CAE patients that was similar to healthy controls. However, the strength, efficiency and small-world properties of the structural network in CAE were seriously damaged. At the whole brain level, decreased strength, global efficiency, local efficiency, clustering coefficient, normalized clustering coefficient and small-worldness values of the network were detected in CAE, while the values of characteristic path length and normalized characteristic path length were abnormally increased. At the regional level, especially the prominent regions of the bilateral precuneus showed reduced nodal efficiency, and the reduction of efficiency was significantly correlated with disease duration. The current results demonstrate significant alterations of structural networks in CAE patients, and the impairments tend to grow worse over time. Our findings may provide a new way to understand the pathophysiological mechanism of CAE.

## Introduction

Childhood absence epilepsy (CAE) is a childhood epilepsy syndrome occurring in 10–17% of all childhood onset epilepsy, making it the most common epilepsy syndrome in school-aged children^1^. In the ILAE definition, CAE is characterized by frequent and transient impairment of consciousness (with abrupt onset and offset) and accompanied with bilateral, symmetrical, and synchronous discharges of 3 Hz generalized spike-and-waves on electroencephalography (EEG)^2^. Although labelled “benign”, a broad spectrum of comorbidities, such as cognitive, behavioural and emotional disorders as well as linguistic deficits have been reported in CAE^3–5^. Moreover, the latest report from the Childhood Absence Epilepsy Study Group even showed obesity and overweight as CAE comorbidities^6^. All these comorbidities and the epilepsy itself can seriously affect children’s lives, making it of great importance to pay more attention to this disorder.

The human brain has been confirmed to be a large-scale complex network^7,8^. Recently, an increasing number of brain analyses based on modern brain mapping techniques (especially, fMRI) have indicated that CAE might be a disorder of brain networks that are crucial for spike-and-wave discharge (SWD) generation and propagation rather than some isolated brain areas^9–11^. Abnormal functional networks have been uncovered in CAE based on fMRI, such as the attention network^12,13^ and the salience network^14^. Moreover, the combination of fMRI and graph theoretical analysis provides an optimal approach to quantifying topological and organizational properties of complex networks in brain disorders^15,16^. Indeed, an adaptive reconfiguration of whole-brain functional network topology has been observed during absence seizure using graph theoretical analysis^10^. In graph theory, a graph G is defined by a set of nodes and edges, where nodes represent the brain regions and edges represent connections between these regions. Despite these advances in research, the structural basis underlying these functional networks of CAE was poorly understood.

Diffusion tensor imaging (DTI) is a non-invasive technique that can be used to explore structural characteristics of brain networks^17^, and Xue *et al*. tried to investigate the whole brain structural network of CAE by parcelling the brain into 90 regions based on an automated anatomical labelling (AAL) atlas^18,19^. Indeed, altered whole-brain structural network topology (e.g., decreased strength, clustering coefficient and efficiency and increased characteristic path length) and impaired sub-networks in sub-cortical and orbitofrontal structures were found in their study. Unfortunately, that remains the only study concerning the topological architecture of structural brain networks of CAE at present, and the fibre assignment by continuous tracking (FACT) algorithm, employed in their study during the process of network construction, was still under heavy debate regarding the fibre-crossing problem within per-voxel. In addition, it still remains unclear as to whether altered topological organization of modular and hub distribution of structural brain networks exists in CAE, which has been suggested to be highly associated with brain dysfunction in other epilepsies^20,21^. Another DTI study concerning CAE also observed some microstructural changes^22^. However, they only explored alterations of the genu of the corpus callosum without further investigation of the whole brain network.

In the current study, we used diffusion probabilistic tractography^23^ and graph theoretical analysis to examine the topological characteristic of structural networks in CAE compared with age- and gender-matched healthy controls. Previous studies have suggested that structural connections are highly mirrored by and place constraints on functional interactions across brain networks^24,25^. Accordingly, we expected to confirm a disrupted structural network in CAE as revealed by the previous research^18^. Moreover, a further exploration of hub and modular topology was conducted in the present study. Specifically, we focused on the differences of network efficiency, modularity and small-worldness between two groups at the global level. At the regional level, we mainly focused on the distribution of hubs identified by nodal efficiency, which is a direct index reflecting the connectivity between a certain node and all the other nodes. In addition, correlations between network metrics and clinical characteristics were performed.

## Results

### Network strength, efficiency and small-world properties

**Figure 1:**
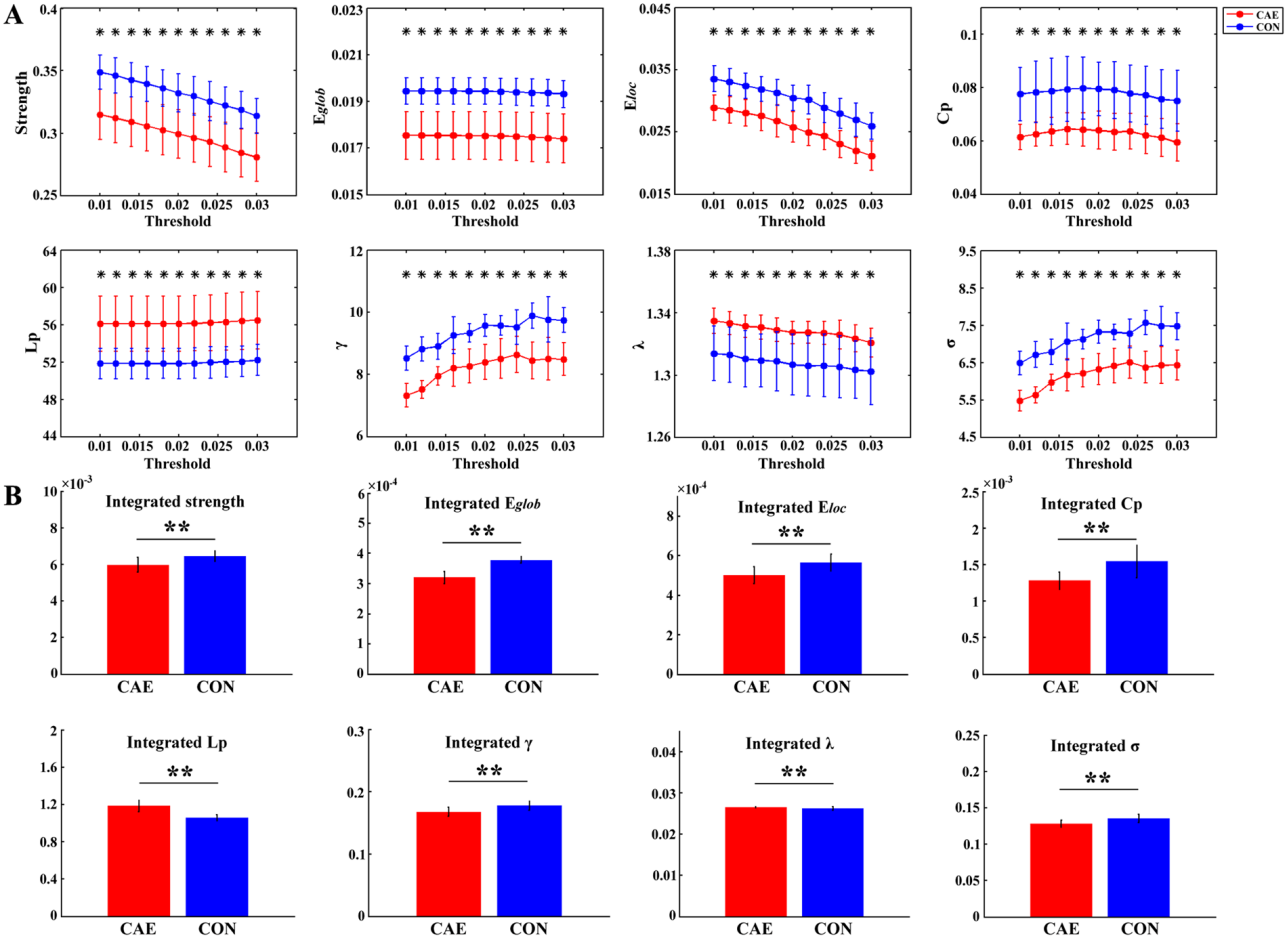
Differences of network metrics between CAE patients and healthy controls (CON). (**A**) Shows the group differences under different thresholds; significant between-group differences are indicated by an asterisk above the corresponding threshold at *p* < 0.05 with Bonferroni correction. (**B**) Displays the group differences at integrated level (**p < 0.05). The error bar indicates standard deviation.

As shown in Fig. 1, CAE patients are found to have decreased strength, *E*_*glob*_, *E*_*loc*_, *C*_*P*_, *γ* and *σ* and increased *L*_*P*_ and *λ* when compared to controls. The between-group differences of these network properties are statistically significant at both the single threshold level and integrated level (Fig. 1). For details, differences of network parameters at all thresholds from 0.01 to 0.03 are presented in Fig. 1A (*p* < 0.05, Bonferroni correction), and differences of integrated values of network parameters are displayed in Fig. 1B.

### Network modularity

**Figure 2:**
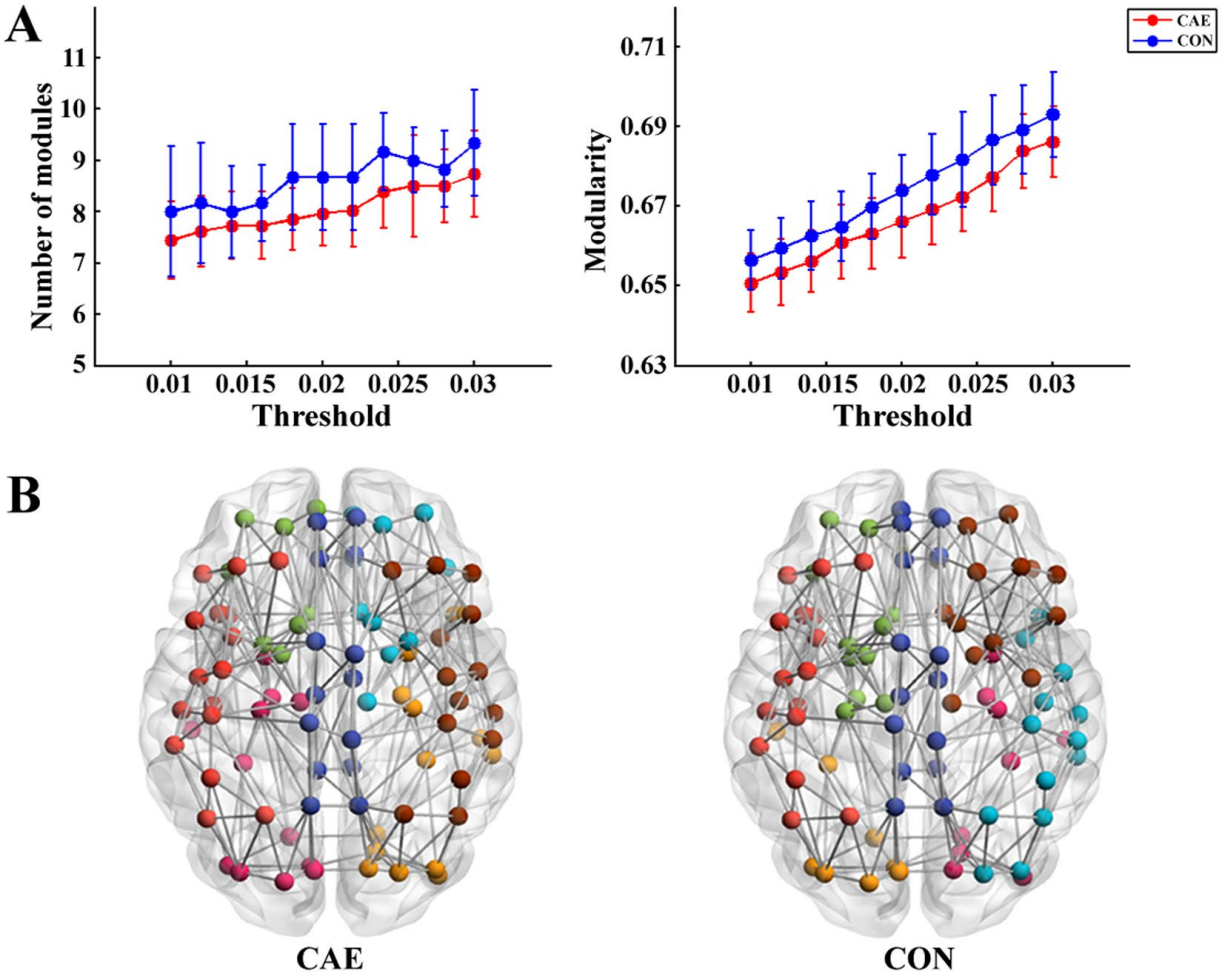
Modular topology of the structural network of CAE patients and healthy controls (CON). (**A**) Number of modules and brain network modularity in CAE and CON under different thresholds. No significant difference is detected between the two groups. The error bar indicates standard deviation. (**B**) 3D representations of modular topology are visualized on the average structural network of each group. The nodes are colour-coded by modules.

The structural brain networks of both patients and controls have a modular community structure (see Supplementary Table S2 for details). The modularity and number of modules of all participants are shown in Fig. 2A. Although a lower number of modules and modularity index are seen in the CAE patients compared with controls at all thresholds, all these between-group differences are not statistically significant. Figure 2B shows a 3D representation of the modular configurations of the average network of each group, and the modular distributions of CAE and controls seem relatively similar.

### Hubs

**Figure 3:**
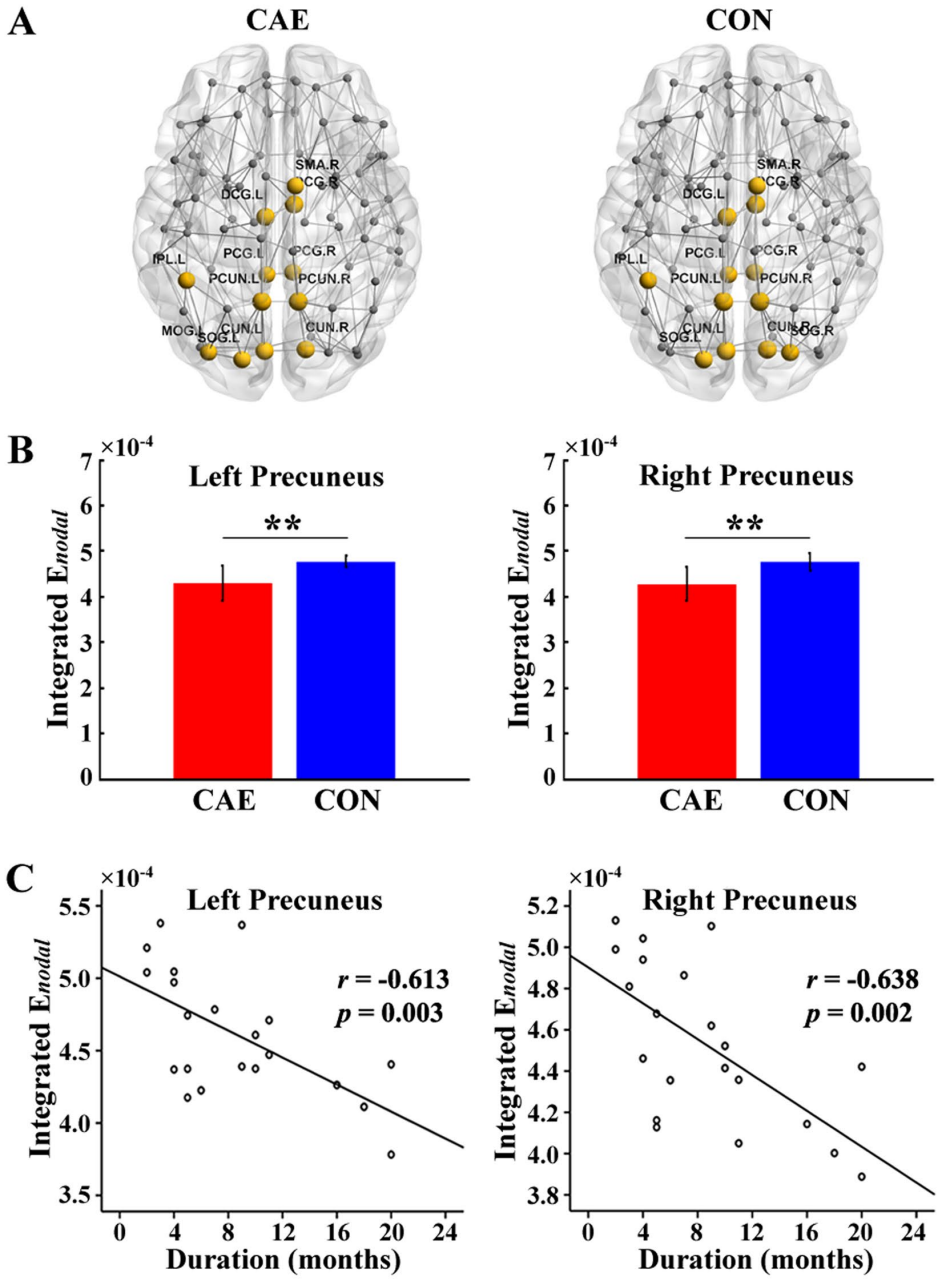
(**A**) Distribution of hubs in CAE and healthy controls (CON). For abbreviations of the regions, please see Supplementary Table S1. (**B**) Significant group difference in integrated E_*nodal*_ of bilateral precuneus (left and right, respectively) (**p < 0.05). (**C**) Relationship between disease duration and integrated E_*nodal*_ of bilateral precuneus in the patient group. Significantly negative correlations are revealed for both sides.

Hub distributions in patients and controls can be seen in Fig. 3A. The definition of hub follows the criterion of ${E}_{nodal}(i) > mean+SD$ (for details, please see Supplementary materials). We found that hubs for CAE and controls are relatively similar. The nodes of bilateral precuneus, bilateral median cingulate and paracingulate gyri, bilateral cuneus, bilateral posterior cingulate gyrus, right supplementary motor area, left superior occipital gyrus and left inferior parietal gyrus are identified as hubs for both patients and controls. A further analysis of the bilateral precuneus was performed since it got the maximum nodal efficiency among all 90 nodes in both groups (Supplementary Fig. S1). Figure 3B shows that the AUC of nodal efficiency of the bilateral precuneus is obviously decreased in CAE compared with controls. Correlation analysis between integrated *E*_*nodal*_ and clinical data (disease duration and seizure frequency) shows a significant correlation between the integrated *E*_*nodal*_ of the bilateral precuneus and disease duration (Fig. 3C), while no significant correlation was detected between the integrated *E*_*nodal*_ of the bilateral precuneus and seizure frequency (Supplementary Fig. S2).

## Discussion

To the best of our knowledge, this is the first study to explore topological alterations of structural networks in CAE patients using probabilistic tractography and graph theory. We have discovered statistically significant differences in network patterns between CAE and controls. Strength, *E*_*glob*_, *E*_*loc*_, *C*_*P*_, *γ* and *σ* are significantly reduced in patients compared with controls, while *L*_*P*_ and *λ* are increased in CAE. Module and hub distributions are relatively similar between the groups.

The decrease of network strength, global efficiency and local efficiency detected in our study indicates a sparser or less efficient network in CAE. This weakness shift of the brain networks in CAE has been consistently reported in a previous study^18^.

The ‘small-world’ organization network is featured by a combination of high *C*_*P*_ and short *L*_*P*_, corresponding to an intermediate state between that of random and regular networks^26^. Originally detected in social networks, the small-world property has been extensively reported in human brain networks in either health or disease^27^. In this paper, small-world topology is revealed in both groups with their small-worldness index *σ* following the criterion (*σ* > 1) under all thresholds^28^. However, in contrast to Xue *et al*.^18^, a further discovery observed in our study is that small-world metrics (including *γ*, *λ* and *σ*) differed significantly. This discrepancy might be attributed to the different tractography algorithm as mentioned in the Introduction section and some other factors (e.g., the data themselves). Specifically, *σ* is an integrated measure that quantifies small-world networks^28^, and a lower value of *σ* indicates damage to small-world topology in CAE patients. The normalized clustering coefficient (*γ*), defined as the ratio of the clustering coefficient of the real network to that of matched random networks, is a good estimation of local network segregation. The lower *γ* implies decreased segregation and a weaker local information processing capacity, which can also be reflected by reduced local efficiency as discussed above. Compared to healthy controls, patients with CAE exhibit reduced *γ* and *σ*, suggesting that the network configuration shifts towards a random network organization. Nevertheless, the value of *λ*, defined as the ratio of the characteristic path length of the real network to that of matched random networks, is significantly increased, indicating less efficiency and higher cost of message transmission along the global network of CAE. This alteration is in the opposite of a randomization shift of a network because a random network is characterized by both low *C*_*P*_ and short *L*_*P*_. There is currently no reasonable explanation for this inconsistency, and thus some further studies on this topic are advisable. Considering the possible effects of normalization on the significance of our results, we also compared the non-normalized form of the above two properties (namely, *L*_*P*_ and *C*_*P*_) between groups, and their results agree well with the properties after normalization (namely, *γ* and *λ*).

Modularity is a frequently used measure of complex networks^29^. Although, numerous fMRI studies have confirmed the existence of modular organization in the human brain^30,31^, no studies have specifically focused on the modularity of CAE patients. Our research reports the presence of modular structure in patients with absence epilepsy for the first time. The modularity index and number of modules of CAE detected in our study exhibit a reduction tendency compared with healthy controls. However, this trend is not statistically significant, and it might be contributing to slight reorganization of modular structure without causing obvious alterations.

In our research, CAE patients and healthy controls demonstrate relatively similar patterns of hub distribution, mainly located in the midline structure of the brain for both groups (Fig. 3A). In brain networks, regions with high levels of centrality can be identified as network hubs using various graph measures (e.g., nodal degree, betweenness and efficiency)^32^. For this study, nodal efficiency is employed, which scales the average shortest path length between the given node and all the other nodes in the network^33^. The nodal efficiency of 90 brain regions according to the AAL atlas is shown in Supplementary Figure S1, and the hub regions are highlighted. Among all the hub nodes, the prominent structural role of the PCUN deserves particular note. It is involved in the process of visuospatial imagery and episodic memory retrieval^34^. Moreover, a clinical review has confirmed that deactivation of the PCUN is associated with loss of consciousness^35^, which is the typical feature of absence seizure. In our study, reduced nodal efficiency of the bilateral PCUN is detected in CAE (Fig. 3B), providing a structural basis for PCUN dysfunctions in absence epilepsy revealed by fMRI^36,37^ and high-frequency magnetoencephalography^38^. In addition, correlation analysis shows a significantly negative correlation between disease duration and PCUN nodal efficiency for both sides (Fig. 3C) that predicts the longer the duration, the worse the impairment. Even though whether the structural-functional impairments are reasons or consequences of absence seizure remains unknown so far, we tentatively support the hypothesis that impairments may be the result of long-term and reiterative epileptic discharges or at least aggravated by chronic absence seizure. Moreover, the alterations of the PCUN further confirm the vulnerability of hub nodes in brain diseases^39^. However, no obvious correlation is observed between PCUN nodal efficiency and seizure frequency (Supplementary Fig. S2). The first explanation for this phenomenon can be attributed to the interictal discharges that certainly affect the brain’s topology without arousing clinical seizure. Additionally, missing records should be taken into account as typical absence seizure is characterized by transient loss of consciousness unlike tonic-clonic seizures, and many absence seizures often occur during sleep. Furthermore, the PCUN is a core area in the default mode network (DMN)^40^, and damage to the PCUN may suggest involvement of the DMN in the neuro-pathophysiological mechanism of CAE, which has been widely discussed in other diseases^41,42^.

### Limitations

Although our methodology yields promising results, there are several methodological issues in our case. First, the probabilistic tractography used in our study has shown advantages in solving the problem of fibre crossing^23^, but it also leads to a new problem of spurious connections that do not exist in the real brain network at the same time. Even with a suggested a thresholding procedure^43^, we may also miss some biological connections or include spurious connections for there is currently no definitive way to select thresholds. Second, there has been no consensus on the definition of edge weight in DTI network analysis. In addition to the connectivity probability, both the average FA of all the voxels along the fibres connecting two regions and the fibre number connecting two regions have been widely employed as the weight of an edge^44–46^. The various definitions of network weight may dramatically affect the precision of calculating network properties. Hence, a further study concerning CAE with comparisons among different definitions of network weight is necessary. The third issue relates to the variance of antiepileptic drugs in our study, which may somehow disturb the results. For instance, valproic acid, which is one of the most common treatments for CAE, has been concluded to be associated with cerebral atrophy in some studies^47,48^. Finally, although a broad spectrum of cognitive and emotional comorbidities have been reported in children suffering from absence epilepsy^5^, we did not include neuropsychological test scores of CAE in this study, which will be assessed in subsequent work.

## Conclusions

In conclusion, we use probabilistic tractography and graph theory to demonstrate that CAE patients display prominent small-world properties close to that of the brain network of healthy controls, uncovering the existence of modular topology in CAE patients for the first time. More importantly, our results provide experimental support for the impaired organization of structural brain networks in CAE, especially in the prominent regions of the bilateral precuneus, the nodal efficiency of which is significantly correlated with disease duration. The topological reconfiguration of structural networks may not only offer further support for dysfunctions in fMRI studies but also contribute to reveal the pathophysiological mechanism of CAE.

## Material and Methods

### Subjects

We recruited 21 patients (8 M/13 F, age range from 5 to 11 years old with mean and standard deviation of 8.05 ± 1.99) and 24 age-and gender-matched healthy controls (11 M/13 F, age range from 5 to 12 years old with mean and standard deviation of 7.63 ± 2.12). There is no significant difference in age (*p* = 0.50, t-test) or gender (*p* = 0.76, chi-square test) between the two groups (see Table 1 for details). All participants in our study are right-handed. CAE was diagnosed according to the guidelines provided by the International League Against Epilepsy^2^. Patients were selected based on the following criteria: (1) routine video-EEG showing symmetric and synchronous generalized SWDs at approximately 3 Hz accompanied by clinical absence seizure; (2) normal neurological and general physical examination; (3) normal structural imaging at 3.0 T MRI scanner.

**Table 1:** Demographic and clinical data of patients.

Subjects ID	Sex	Age (y)	Disease duration (m)	Frequency of seizure (times/d)	AED treatment	Frequency of SWDs (Hz)
1	M	10	20	7	VPA	2–3
2	M	8	4	4	VPA	2.5–3
3	M	7	7	10	None	3
4	F	9	9	6	LEV	3
5	M	8	5	16	VPA	3–3.5
6	F	7	5	6	VPA	3
7	F	9	6	1	None	2–3
8	M	6	4	5	VPA	3
9	F	7	4	14	VPA	3
10	F	9	10	3	LEV	3
11	M	5	5	7	VPA	3
12	F	8	18	3	None	3–3.2
13	M	10	9	5	LEV	3
14	M	11	15	16	VPA, LEV	3
15	M	8	10	4	VPA	2.5–3
16	F	9	11	4	LEV	3
17	M	9	11	6	VPA	2.5–3.5
18	F	6	2	13	LEV	3
19	F	11	20	9	None	2.5–3.5
20	F	10	2	15	LEV	3
21	F	6	3	9	VPA	3

M = male; F = female; d = day; m = months; y = years; AED = antiepileptic drug; LEV = levetiracetam; VPA = valproic acid; SWDs = spike wave discharges.

This study was approved by the ethical boards of Nanjing Medical University. Written informed consent was obtained from the parents of each of the children participating in the research.

### Image acquisition

All MRI data were acquired on a Siemens 3.0 T scanner (Erlangen, Germany). Diffusion weighted images were acquired using a single-shot echo planar imaging sequence with the following parameters: 45 axial slices; slice thickness = 3 mm with no gap; repetition time (TR) = 6600 ms; echo time (TE) = 93 ms; 30 diffusion directions with b = 1000 s/mm^2^; and an additional image without diffusion weighting (b = 0 s/mm^2^); acquisition matrix = 128 × 128; field of view (FOV) = 240 × 240 mm^2^. For the process of segmentation and registration, high-resolution T1-weighted MRI was also obtained using a three-dimensional rapid acquisition gradient echo sequence with the following parameters: 176 sagittal slices; slice thickness = 1 mm; TR = 1900 ms; TE = 2.48 ms; flip angle = 9°; acquisition matrix = 512 × 512, FOV = 250 × 250 mm^2^.

To minimize head motion, participants were instructed to lie down in the supine position with their head restrained by foam pads during scanning.

### Data processing and network construction

**Figure 4:**
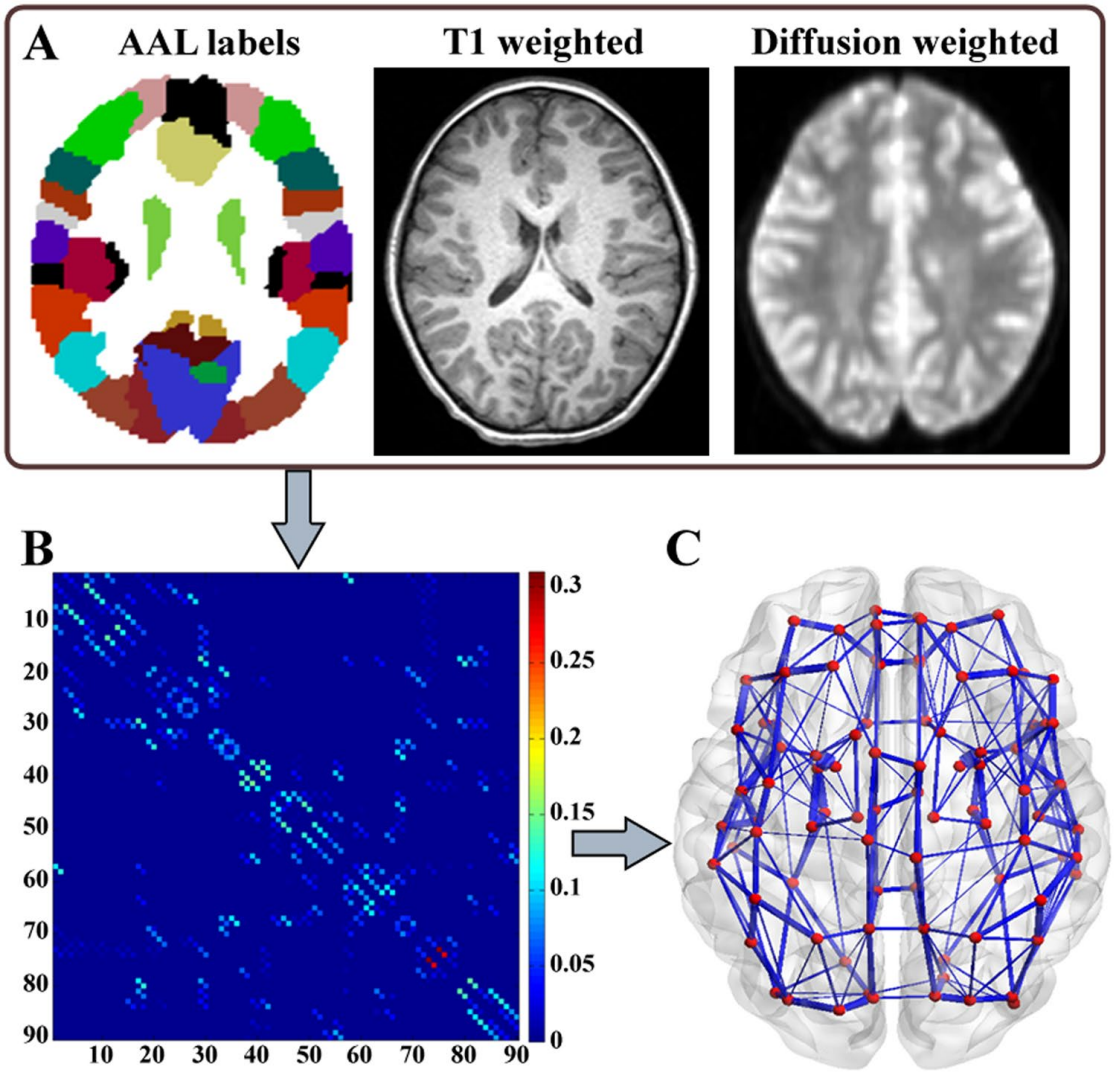
Flowchart for brain network construction. Data from a typical healthy control is used to demonstrate the process of construction. (**A**) Raw high-resolution T1-weighted MRI and DTI images in native space and automated anatomical labelling (AAL) atlas in MNI space. (**B**) The connectivity matrix is built up according to the probabilistic tractography algorithm after the process of parcellation and normalization. (**C**) 3D visualization of the weighted network is rendered using BrainNet Viewer (BrainNet Viewer 1.53, Beijing Normal University, http://www.nitrc.org/projects/bnv/)^55^. The edges are encoded with their connection weights at the threshold of 0.01.

Processing and analysis of the DTI data were performed in FSL (FSL 4.1, FMRIB’s Software Library, www.fmrib.ox.ac.uk/fsl). The preprocessing procedure included eddy-current and head-motion correction. To address concerns about potential confounding effects of head motion on diffusion metrics, a single value, the total motion index (TMI)^49^ was calculated, and no significant difference in TMI between CAE patients ([mean ± SD]: 0.407 ± 1.199) and healthy controls ([mean ± SD]: 0.252 ± 0.980) (*p* = 0.637) was detected. After fitting a tensor model to the diffusion-weighted images tensor, voxel-wise measures of fractional anisotropy (FA) images were calculated. All these steps were done using FMRIB’s Diffusion Toolbox. In addition, non-brain tissues were extracted using the Brain Extraction Toolbox (BET) implemented in FSL^50^. Then, a network composed of 90 nodes and a quantity of edges linking these nodes for each subject was constructed following the workflow in Fig. 4, including brain parcellation (Fig. 4A) and a connectivity matrix resulting from probabilistic tractography (Fig. 4B). Finally, a 3D visualization of the network was rendered in Fig. 4C.

### Brain parcellation and network node definition

To avoid bias from subjective judgements regarding anatomical correspondences, the Automated Anatomical Labelling (AAL) atlas^19^ was used for network node definition. First, T1-weighted images were co-registered to the b0 images in DTI space using a linear transformation. Next, the co-registered T1-weighted image was normalized to the T1 template in MNI space. Finally, inverse transformations were applied to warp the AAL atlas from the MNI space to the native diffusion space. Of note, discrete labelling values were preserved by the use of a nearest-neighbour interpolation method. All the works above were completed using SPM12 (Statistical Parametric Mapping, http://www.fil.ion.ucl.ac.uk/spm/software/spm12/) running in MATLAB R2012b (Math Works, Natick, MA, USA). As a result, 90 cortical and subcortical regions (45 for each hemisphere), each of which represented a network node in graph theory, were obtained.

### DTI tractography and network edge definition

Probabilistic fibre tracking was performed in native space using the FSL Diffusion Toolbox (FDT) as in previous studies^43,51^. For each defined node, the connectivity probability to each of the other 89 nodes was computed. As a result, a 90 × 90 network matrix *N* was generated, for element *N*_*ij*_ means the connectivity probability from node *i* to node *j*. However, because of the tractography’s dependence on the seeding location, the probability from *i* to *j* did not exactly equal that from *j* to *i*. Therefore, we averaged the connectivity probability from *i* to *j* with that from *j* to *i*, and defined the average connectivity probability as the connection weight between node *i* and *j*, *w*_*ij*_ = (*N*_*ij*_ + *N*_*ji*_)/2. Then, we got a symmetrical 90 × 90 connection matrix, and a weighted network was also constructed for each individual participant.

### Network properties

A brain network can be regarded as a graph G that consists of a set of nodes and a set of edges connecting these nodes^15^. For complex networks, the strength, global efficiency (*E*_*glob*_), local efficiency (*E*_*loc*_), clustering coefficient (*C*_*P*_), characteristic path length (*L*_*P*_), normalized clustering coefficient (*γ*), normalized characteristic path length (*λ*), small-worldness (*σ*), modularity of the network and nodal efficiency (*E*_*nodal*_) are commonly used metrics in network topological analyses. Brief descriptions of these parameters are addressed in Supplementary materials, and the mathematical definitions can be found in a previous study^52^. After acquiring the weighted matrix of a network, these properties can be directly calculated with the Brain Connectivity Toolbox^52^ in MATLAB R2012b. It should be noted that there is currently no criterion for selecting a single threshold to construct structural brain networks. For this reason, the individual weighted matrices were repeatedly analysed over a range of connectivity thresholds (ranging from 0.01 to 0.03 with an interval of 0.002, all 11 thresholds), where a threshold indicates that only weights higher than the given threshold are preserved and others are ignored in the weighted matrix. Afterwards, the area under curve (AUC) of each metric over the range of thresholds (0.01~0.03) was calculated for each subject, which is an integrated scalar for the topological characterization of brain networks and independent of a single threshold selection. These integrated metrics have also been employed in recent brain network research^53,54^.

### Statistical Analysis

Two-sample t-tests were repeatedly performed for differences of network properties between the CAE patients and healthy controls. The relationships between network measures and clinical characteristics of patients were analysed using Pearson’s correlation. All statistical analyses were performed in Matlab R2012b with the Statistics Toolbox and some custom scripts. Differences were accepted as significant at the level of 0.05 for each comparison. For multiple comparisons under 11 thresholds, a *p*-value < 0.05/11 was considered significant with Bonferroni correction.

## Electronic supplementary material

Supplementary Information
