# Supplementary material for: Disrupted topological organization of structural brain networks in childhood absence epilepsy
Source: Sci Rep. 2017 Sep 20;7:11973. doi: 10.1038/s41598-017-10778-0 (PMC5607318; doi:10.1038/s41598-017-10778-0)
Supplement: Supplementary file 1 — Supplementary Information [file 41598_2017_10778_MOESM1_ESM.pdf]

# Disrupted topological organization of structural brain networks in childhood absence epilepsy

Wenchao Qiu[1], Chuanyong Yu[1], Yuan Gao[1], Ailiang Miao[1], Lu Tang[1], Shuyang Huang[1], Wenwen Jiang[1], Jintao Sun[1], Jing Xiang[2], Xiaoshan Wang[1*]

1 Department of neurology, Nanjing Brain Hospital, Nanjing Medical University, China.
2 Division of Neurology, Cincinnati Children's Hospital Medical Center, USA

*corresponding author:
Xiaoshan Wang,
Institute: Department of Neurology, Nanjing Brain Hospital, Nanjing Medical University, Nanjing, Jiangsu, China.
Address: Guang Zhou Road 264, Nanjing, Jiangsu 210029, China.
Fax: +86 25 8371 9457.
E-mail：873734205@qq.com.

**Supplementary Materials**

**Strength**

Strength of node ***i*** is defined as the sum of edge weights linking to it [1], while network strength means the average of the strengths across all the nodes.

$$S(\mathrm{G}) = \frac{1}{N}\sum_{i \in G} S_i$$

Where $N$ is the number of nodes in graph (network) $G$ and $S_i$ is the sum of the edge weights $w_{ij}$ connecting to node ***i***.

**Efficiency**

Efficiency of network, including global and local efficiency, is a measure of how efficiently it exchanges information in the network [2]. The global efficiency ($E_{glob}$) represents the capability of information flow over the entire network. In this work, we defined the global efficiency using Dijkstra's algorithm [3,4].

$$E_{glob}(\mathrm{G}) = \frac{1}{N(N-1)}\sum_{i \neq j \in G} \frac{1}{L_{ij}}$$

Where $L_{ij}$ is the shortest path length between node ***i*** and node ***j*** in ***G***.

The local efficiency ($E_{loc}$) indicates how well the information is communicated within the neighbors of a given node. Dijkstra's algorithm was also employed to calculate the

local efficiency [3,4].

$$E_{loc}(\mathrm{G}) = \frac{1}{N}\sum_{i\in G} E_{glob}(G_i)$$

Where $\boldsymbol{E_{glob}(G_i)}$ is the global efficiency of $\boldsymbol{G_i}$, the subgraph of the neighbors of node $\boldsymbol{i}$.

**Small-worldness**

Small-world measures, including clustering coefficient ($\boldsymbol{C_P}$), characteristic path length ($\boldsymbol{L_P}$), normalized clustering coefficient ($\boldsymbol{\gamma}$), normalized characteristic path length ($\boldsymbol{\lambda}$), and small-worldness ($\boldsymbol{\sigma}$), are most frequently used properties in brain network study [5]. Highly interconnected neighbors around a given node form a cluster, while sparsely interconnected neighbors do not. Clustering coefficient of node $\boldsymbol{i}$ ($\boldsymbol{C_i}$) reflects the number of connections among the neighbors of node $\boldsymbol{i}$.

$$C_i = \frac{2t_i}{k_i(k_i - 1)}$$

Where $\boldsymbol{k_i}$ is the degree of node $\boldsymbol{i}$ and $\boldsymbol{t_i}$ is the number of triangles around node $\boldsymbol{i}$.

Moreover, the clustering coefficient of a network ($\boldsymbol{C_P}$) is quantified as the average of the clustering coefficient over all nodes, which characterizes network segregation.

$$C_P(\mathrm{G}) = \frac{1}{N}\sum_{i\in G} C_i$$

Characteristic path length ($\boldsymbol{L_P}$) is defined as the average of shortest path length between all nodal pairs, which characterizes the integration or information transfer capacity across remote cortical regions.

$$L_P = \frac{1}{N(N-1)}\sum_{i\neq j\in G} L_{ij}$$

Where $\boldsymbol{L_{ij}}$ is the shortest path length from node $\boldsymbol{i}$ to node $\boldsymbol{j}$.

Moreover, the normalized clustering coefficient ($\boldsymbol{\gamma}$) and the normalized characteristic path length ($\boldsymbol{\lambda}$) were obtained from comparing $\boldsymbol{C_P}$ and $\boldsymbol{L_P}$ of brain network with that of 100 random networks with the same number of nodes and degree distribution.

$$\gamma = \frac{C_P}{C_P^{rand}}, \lambda = \frac{L_P}{L_P^{rand}}$$

Where $\boldsymbol{C_P^{rand}}$ and $\boldsymbol{L_P^{rand}}$ are the mean of $\boldsymbol{C_P}$ and $\boldsymbol{L_P}$ of 100 matched random networks. The brain network would be considered as small-world if $\gamma \gg 1$ and $\lambda \approx 1$. Furthermore, these two properties can be summarized into a simple quantitative metric, small-worldness ($\boldsymbol{\sigma}$):

$$\sigma = \frac{\gamma}{\lambda}$$

which is higher than 1 for the small-world network [6].

**Hubs**

Important brain regions, known as hubs, often communicates more efficiently with the rest of the brain [7,8]. To determine the hubs distribution, we computed the nodal efficiency, $\boldsymbol{E_{nodal}(i)}$, and define node i as a network hub if $\boldsymbol{E_{nodal}(i)}$ follows the criterion:

$E_{nodal}(i) > mean + SD$ [9]. Nodal efficiency measures the average shortest path length between a given node and all of the other nodes in the network.

$$E_{nodal}(i) = \frac{1}{N-1} \sum_{i \neq j \in G} \frac{1}{L_{ij}}$$

Where $L_{ij}$ is defined as the length of shortest path between node $i$ and node $j$.

**Modularity**

Modular structure is consist of densely interconnected nodes that have only sparse interconnections with others. The modularity index $Q$ is used to estimate the degree how the network may be divided into such subgroups [10].

$$Q = \frac{1}{l} \sum_{i,j \in G} \left( w_{ij} - \frac{k_i k_j}{l} \right) \delta_{m_i, m_j}$$

Where $l$ is the sum of all weights in the network, $w_{ij}$ is the edge weight between node $i$ and node $j$ and $k_i$ is the degree of node $i$. Note that $m_i$ is the module containing node $i$, and $\delta_{mi,mj}$ =1 if $m_i = m_j$ and 0 otherwise.


1 Barrat, A., Barthelemy, M., Pastor-Satorras, R. & Vespignani, A. The architecture of complex weighted networks. *Proceedings of the National Academy of Sciences of the United States of America* **101**, 3747-3752, doi:10.1073/pnas.0400087101 (2004).

2 Achard, S. & Bullmore, E. Efficiency and cost of economical brain functional networks. *PLoS Comput. Biol.* **3**, e17, doi:10.1371/journal.pcbi.0030017 (2007).

3 Onnela, J. P., Saramaki, J., Kertesz, J. & Kaski, K. Intensity and coherence of motifs in weighted complex networks. *Physical review. E, Statistical, nonlinear, and soft matter physics* **71**, 065103, doi:10.1103/PhysRevE.71.065103 (2005).

4 Latora, V. & Marchiori, M. Efficient behavior of small-world networks. *Physical review letters* **87**, 198701, doi:10.1103/PhysRevLett.87.198701 (2001).

5 Watts, D. J. & Strogatz, S. H. Collective dynamics of 'small-world' networks. *Nature* **393**, 440-442, doi:10.1038/30918 (1998).

6 Humphries, M. D. & Gurney, K. Network 'small-world-ness': a quantitative method for determining canonical network equivalence. *PLoS One* **3**, e0002051, doi:10.1371/journal.pone.0002051 (2008).

7 Sporns, O., Honey, C. J. & Kotter, R. Identification and classification of hubs in brain networks. *PLoS One* **2**, e1049, doi:10.1371/journal.pone.0001049 (2007).

8 van den Heuvel, M. P. & Sporns, O. Network hubs in the human brain. *Trends in cognitive sciences* **17**, 683-696, doi:http://dx.doi.org/10.1016/j.tics.2013.09.012 (2013).

9 Cao, Q. *et al.* Probabilistic diffusion tractography and graph theory analysis reveal abnormal white matter structural connectivity networks in drug-naive boys with attention deficit/hyperactivity disorder. *The Journal of neuroscience : the official journal of the Society for Neuroscience* **33**, 10676-10687, doi:10.1523/jneurosci.4793-12.2013 (2013).

10 Newman, M. E. Modularity and community structure in networks. *Proceedings of the National*

*Academy of Sciences of the United States of America* **103**, 8577-8582, doi:10.1073/pnas.0601602103 (2006).

**Supplementary Figure S1**

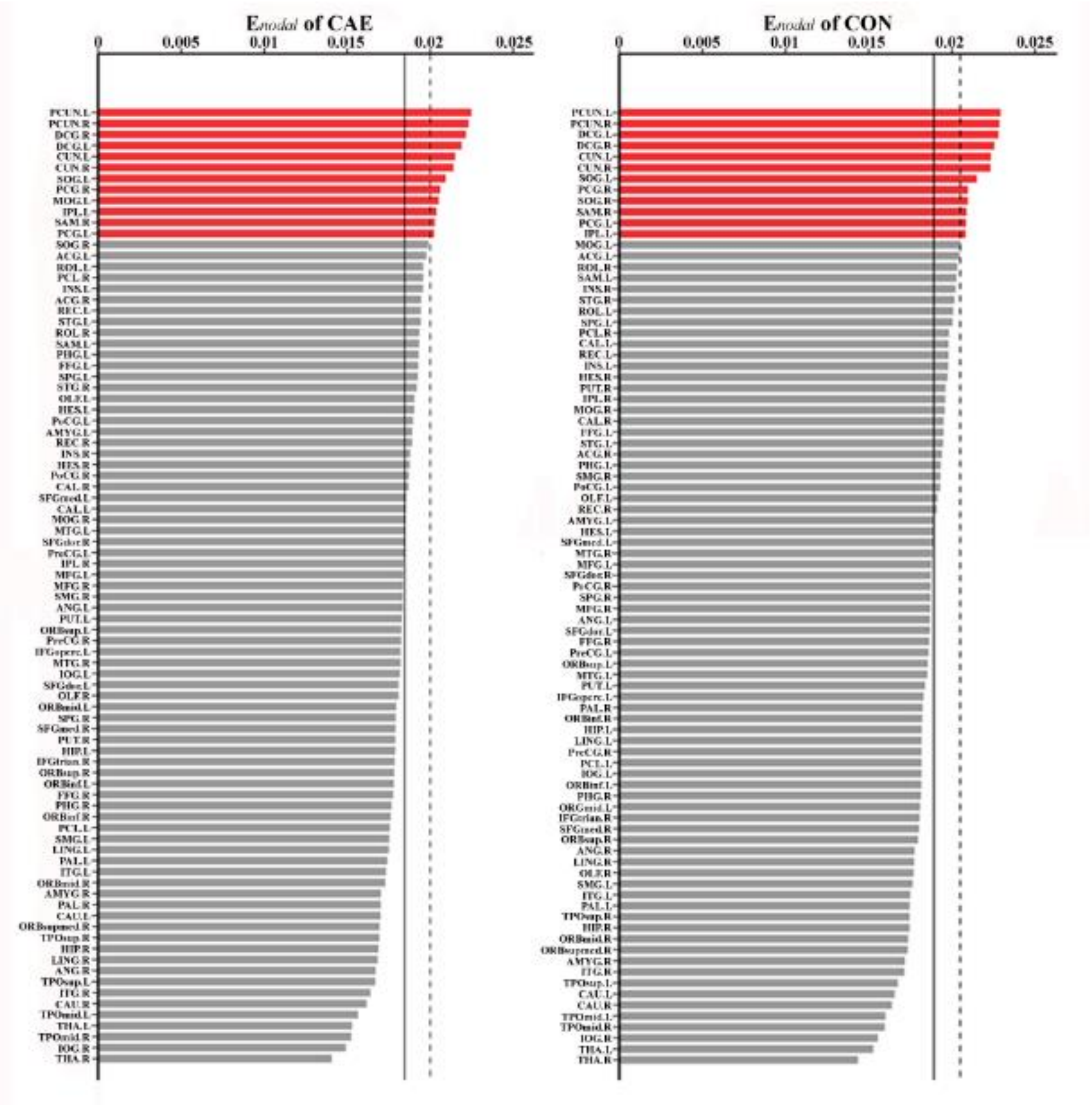


Nodal efficiency (E*nodal*) of 90 regions in CAE and healthy controls (CON). The E*nodal* of each region is calculated from the average network of each group. Areas are ranked in descending order. The solid line marks the mean E*nodal* and dashed line marks the mean plus one standard deviation. Hub regions are highlighted in red based on the criterion of $E_{nodal}(i) > mean + SD$ for both group, while others are labeled in gray. For abbreviations of the regions, please see Supplementary Table S1.

**Supplementary Figure S2**

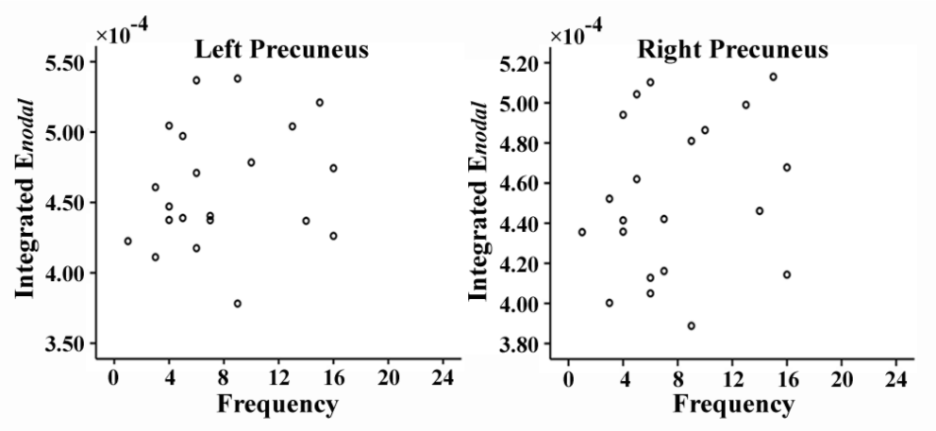


Scatter plots between seizure frequency and integrated $E_{nodal}$ of the left and right precuneus, respectively.

**Supplementary Table S1** Regions of interest (45 in each cerebral hemisphere) defined in this study using AAL atlas.

| Index | Regions | Abbreviation |
|---|---|---|
| 1 | Precentral | PreCG |
| 2 | Superior frontal gyrus (dorsolateral) | SFGdor |
| 3 | Superior frontal gyrus (orbital part) | ORBsup |
| 4 | Middle frontal gyrus | MFG |
| 5 | Middle frontal gyrus (orbital part) | ORBmid |
| 6 | Inferior frontal gyrus (opercular part) | IFGoperc |
| 7 | Inferior frontal gyrus (triangular part) | IFGtriang |
| 8 | Inferior frontal gyrus (orbital part) | ORBinf |
| 9 | Rolandic operculum | ROL |
| 10 | Supplementary motor area | SMA |
| 11 | Olfactroy cortex | OLF |
| 12 | Superior frontal gyrus (medial) | SFGmed |
| 13 | Superior frontal gyrus (medial orbital) | ORBsupmed |

| | | |
|---|---|---|
| 14 | Rectus gyrus | REC |
| 15 | Insula | INS |
| 16 | Anterior cingulate gyri | ACG |
| 17 | Median cingulate gyri | MCG |
| 18 | Posterior cingulate gyrus | PCG |
| 19 | Hippocampus | HIP |
| 20 | Parahippocampalgyrus | PHG |
| 21 | Amygdala | AMYG |
| 22 | Calcarine fissure | CAL |
| 23 | Cuneus | CUN |
| 24 | Lingual gyrus | LING |
| 25 | Superior occipital gyrus | SOG |
| 26 | Middle occipital gyrus | MOG |
| 27 | Inferior occipital gyrus | IOG |
| 28 | Fusiform gyrus | FFG |
| 29 | Postcentralgyrus | PoCG |
| 30 | Superior parietal gyrus | SPG |
| 31 | Inferior parietal gyrus | IPG |
| 32 | Supramarginalgyrus | SMG |
| 33 | Angular gyrus | ANG |
| 34 | Precuneus | PCUN |
| 35 | Paracentral lobule | PCL |
| 36 | Caudate nucleus | CAU |
| 37 | Putamen | PUT |

| | | |
|---|---|---|
| 38 | Pallidum | PAL |
| 39 | Thalamus | THA |
| 40 | Heschlgyrus | HES |
| 41 | Superior temporal gyrus | STG |
| 42 | Superior temporal gyrus, temporal pole | TPOsup |
| 43 | Middle temporal gyrus | MTG |
| 44 | Middle temporal gyrus, temporal pole | TPOmid |
| 45 | Inferior temporal gyrus | ITG |

The abbreviations are listed according to Salvador [11], which differ slightly from the original abbreviations by Tzourio-Mazoyer [12].


1 Barrat, A., Barthelemy, M., Pastor-Satorras, R. & Vespignani, A. The architecture of complex weighted networks. *Proceedings of the National Academy of Sciences of the United States of America* **101**, 3747-3752, doi:10.1073/pnas.0400087101 (2004).

2 Achard, S. & Bullmore, E. Efficiency and cost of economical brain functional networks. *PLoS Comput. Biol.* **3**, e17, doi:10.1371/journal.pcbi.0030017 (2007).

3 Onnela, J. P., Saramaki, J., Kertesz, J. & Kaski, K. Intensity and coherence of motifs in weighted complex networks. *Physical review. E, Statistical, nonlinear, and soft matter physics* **71**, 065103, doi:10.1103/PhysRevE.71.065103 (2005).

4 Latora, V. & Marchiori, M. Efficient behavior of small-world networks. *Physical review letters* **87**, 198701, doi:10.1103/PhysRevLett.87.198701 (2001).

5 Watts, D. J. & Strogatz, S. H. Collective dynamics of 'small-world' networks. *Nature* **393**, 440-442, doi:10.1038/30918 (1998).

6 Humphries, M. D. & Gurney, K. Network 'small-world-ness': a quantitative method for determining canonical network equivalence. *PLoS One* **3**, e0002051, doi:10.1371/journal.pone.0002051 (2008).

7 Sporns, O., Honey, C. J. & Kotter, R. Identification and classification of hubs in brain networks. *PLoS One* **2**, e1049, doi:10.1371/journal.pone.0001049 (2007).

8 van den Heuvel, M. P. & Sporns, O. Network hubs in the human brain. *Trends in cognitive sciences* **17**, 683-696, doi:http://dx.doi.org/10.1016/j.tics.2013.09.012 (2013).

9 Cao, Q. *et al.* Probabilistic diffusion tractography and graph theory analysis reveal abnormal white matter structural connectivity networks in drug-naive boys with attention deficit/hyperactivity disorder. *The Journal of neuroscience : the official journal of the Society for Neuroscience* **33**, 10676-10687, doi:10.1523/jneurosci.4793-12.2013 (2013).

10 Newman, M. E. Modularity and community structure in networks. *Proceedings of the National Academy of Sciences of the United States of America* **103**, 8577-8582, doi:10.1073/pnas.0601602103 (2006).

11 Salvador, R. *et al.* Neurophysiological architecture of functional magnetic resonance images of

human brain. *Cerebral cortex* **15**, 1332-1342, doi:10.1093/cercor/bhi016 (2005).

12 Tzourio-Mazoyer, N. *et al.* Automated anatomical labeling of activations in SPM using a macroscopic anatomical parcellation of the MNI MRI single-subject brain. *NeuroImage* **15**, 273-289, doi:10.1006/nimg.2001.0978 (2002).

**Supplementary Table S2.** Comparisons of modularity between subjects and null network models

| Subjects / Thresholds / Z-scores (*p*-value) | 0.010 | 0.012 | 0.014 | 0.016 | 0.018 | 0.020 | 0.022 | 0.024 | 0.026 | 0.028 | 0.030 |
|---|---|---|---|---|---|---|---|---|---|---|---|
| **CAE01** | 14.8861 (<0.001) | 14.2341 (<0.001) | 11.8811 (<0.001) | 14.1582 (<0.001) | 12.6064 (<0.001) | 12.6872 (<0.001) | 13.1619 (<0.001) | 11.6549 (<0.001) | 11.9421 (<0.001) | 11.2228 (<0.001) | 9.8093 (<0.001) |
| **CAE02** | 12.6428 (<0.001) | 15.5350 (<0.001) | 14.1538 (<0.001) | 13.2236 (<0.001) | 14.4799 (<0.001) | 12.5106 (<0.001) | 14.7112 (<0.001) | 14.5750 (<0.001) | 12.6249 (<0.001) | 12.5663 (<0.001) | 11.6261 (<0.001) |
| **CAE03** | 15.6382 (<0.001) | 15.6247 (<0.001) | 16.1235 (<0.001) | 14.1269 (<0.001) | 12.6855 (<0.001) | 12.6250 (<0.001) | 12.0071 (<0.001) | 11.8194 (<0.001) | 12.2396 (<0.001) | 12.6226 (<0.001) | 12.2584 (<0.001) |
| **CAE04** | 13.2820 (<0.001) | 12.0297 (<0.001) | 13.1439 (<0.001) | 15.1606 (<0.001) | 13.1782 (<0.001) | 13.2922 (<0.001) | 12.2425 (<0.001) | 13.2483 (<0.001) | 13.1975 (<0.001) | 11.8258 (<0.001) | 12.7000 (<0.001) |
| **CAE05** | 15.6664 (<0.001) | 13.1617 (<0.001) | 14.8210 (<0.001) | 11.7734 (<0.001) | 13.5224 (<0.001) | 11.7568 (<0.001) | 13.1436 (<0.001) | 13.6158 (<0.001) | 12.7174 (<0.001) | 11.7928 (<0.001) | 12.8647 (<0.001) |
| **CAE06** | 14.7942 (<0.001) | 14.7874 (<0.001) | 13.7315 (<0.001) | 14.6765 (<0.001) | 14.0181 (<0.001) | 15.2846 (<0.001) | 13.3448 (<0.001) | 14.4094 (<0.001) | 13.6887 (<0.001) | 10.5490 (<0.001) | 11.4871 (<0.001) |
| **CAE07** | 14.3138 (<0.001) | 12.6652 (<0.001) | 13.4151 (<0.001) | 14.1245 (<0.001) | 14.4520 (<0.001) | 12.8406 (<0.001) | 12.4846 (<0.001) | 12.7658 (<0.001) | 11.4862 (<0.001) | 12.0074 (<0.001) | 11.7157 (<0.001) |
| **CAE08** | 15.6657 (<0.001) | 16.9333 (<0.001) | 12.9405 (<0.001) | 14.3676 (<0.001) | 13.8009 (<0.001) | 13.7764 (<0.001) | 14.2974 (<0.001) | 12.5718 (<0.001) | 10.9578 (<0.001) | 10.5498 (<0.001) | 10.2674 (<0.001) |
| **CAE09** | 14.8185 (<0.001) | 14.7373 (<0.001) | 16.4189 (<0.001) | 15.7128 (<0.001) | 15.3443 (<0.001) | 13.2281 (<0.001) | 13.9379 (<0.001) | 13.4299 (<0.001) | 13.7279 (<0.001) | 13.1592 (<0.001) | 10.4463 (<0.001) |
| **CAE10** | 12.7471 (<0.001) | 13.9294 (<0.001) | 12.0021 (<0.001) | 13.5987 (<0.001) | 13.6495 (<0.001) | 12.4759 (<0.001) | 12.8867 (<0.001) | 13.7583 (<0.001) | 11.4752 (<0.001) | 11.1565 (<0.001) | 12.5295 (<0.001) |
| **CAE11** | 13.8052 (<0.001) | 13.0221 (<0.001) | 12.9496 (<0.001) | 12.3027 (<0.001) | 13.3998 (<0.001) | 12.0057 (<0.001) | 11.9089 (<0.001) | 13.1770 (<0.001) | 10.5579 (<0.001) | 10.6222 (<0.001) | 9.1986 (<0.001) |
| **CAE12** | 12.9154 (<0.001) | 12.6505 (<0.001) | 13.3597 (<0.001) | 13.3591 (<0.001) | 12.6806 (<0.001) | 11.0594 (<0.001) | 12.5400 (<0.001) | 12.3284 (<0.001) | 13.1781 (<0.001) | 10.1114 (<0.001) | 11.6108 (<0.001) |
| **CAE13** | 13.354 (<0.001) | 15.4795 (<0.001) | 14.2719 (<0.001) | 13.1574 (<0.001) | 14.3260 (<0.001) | 14.6042 (<0.001) | 14.3237 (<0.001) | 10.8848 (<0.001) | 13.4567 (<0.001) | 12.8811 (<0.001) | 12.7190 (<0.001) |
| **CAE14** | 14.6003 (<0.001) | 14.7825 (<0.001) | 15.1020 (<0.001) | 14.6426 (<0.001) | 12.9238 (<0.001) | 11.4754 (<0.001) | 11.8501 (<0.001) | 10.7686 (<0.001) | 9.6947 (<0.001) | 12.3721 (<0.001) | 9.1354 (<0.001) |
| **CAE15** | 15.8903 (<0.001) | 14.3345 (<0.001) | 12.9407 (<0.001) | 13.2115 (<0.001) | 11.5095 (<0.001) | 12.5954 (<0.001) | 13.0674 (<0.001) | 13.5528 (<0.001) | 11.4392 (<0.001) | 15.7165 (<0.001) | 11.9067 (<0.001) |
| **CAE16** | 13.6907 (<0.001) | 16.6381 (<0.001) | 12.8347 (<0.001) | 15.6629 (<0.001) | 13.5608 (<0.001) | 11.7340 (<0.001) | 11.9153 (<0.001) | 13.4488 (<0.001) | 11.4198 (<0.001) | 14.1151 (<0.001) | 13.2720 (<0.001) |
| **CAE17** | 14.6934 (<0.001) | 13.9103 (<0.001) | 11.6914 (<0.001) | 14.3910 (<0.001) | 12.5668 (<0.001) | 12.7782 (<0.001) | 13.1841 (<0.001) | 13.1926 (<0.001) | 11.8085 (<0.001) | 9.9230 (<0.001) | 12.2226 (<0.001) |

| | | | | | | | | | | | |
|---|---|---|---|---|---|---|---|---|---|---|---|
| **CAE18** | 14.8275 (<0.001) | 13.7263 (<0.001) | 15.4149 (<0.001) | 16.7122 (<0.001) | 14.3449 (<0.001) | 13.5261 (<0.001) | 14.9374 (<0.001) | 14.4229 (<0.001) | 13.7319 (<0.001) | 12.1432 (<0.001) | 11.4563 (<0.001) |
| **CAE19** | 15.4303 (<0.001) | 14.3141 (<0.001) | 12.2401 (<0.001) | 14.2145 (<0.001) | 12.5015 (<0.001) | 12.4154 (<0.001) | 13.1174 (<0.001) | 13.1928 (<0.001) | 12.4202 (<0.001) | 15.6765 (<0.001) | 12.1067 (<0.001) |
| **CAE20** | 14.1261 (<0.001) | 13.4441 (<0.001) | 11.1211 (<0.001) | 13.8782 (<0.001) | 13.1165 (<0.001) | 12.3272 (<0.001) | 12.8919 (<0.001) | 11.5449 (<0.001) | 12.2129 (<0.001) | 11.2348 (<0.001) | 9.7993 (<0.001) |
| **CAE21** | 13.2791 (<0.001) | 11.0298 (<0.001) | 12.8839 (<0.001) | 14.9606 (<0.001) | 13.1672 (<0.001) | 14.2923 (<0.001) | 13.2421 (<0.001) | 12.2983 (<0.001) | 12.6975 (<0.001) | 12.1252 (<0.001) | 11.9201 (<0.001) |
| **CON01** | 14.5022 (<0.001) | 13.2124 (<0.001) | 14.8921 (<0.001) | 14.4662 (<0.001) | 12.1337 (<0.001) | 12.4757 (<0.001) | 12.7502 (<0.001) | 10.4613 (<0.001) | 9.8747 (<0.001) | 10.3735 (<0.001) | 10.1354 (<0.001) |
| **CON02** | 14.1952 (<0.001) | 15.3551 (<0.001) | 12.3113 (<0.001) | 12.2703 (<0.001) | 13.3114 (<0.001) | 11.1834 (<0.001) | 11.5517 (<0.001) | 11.8220 (<0.001) | 11.7166 (<0.001) | 12.3129 (<0.001) | 11.8803 (<0.001) |
| **CON03** | 14.3348 (<0.001) | 14.5834 (<0.001) | 13.7262 (<0.001) | 12.4047 (<0.001) | 13.5436 (<0.001) | 13.3672 (<0.001) | 12.5087 (<0.001) | 13.2625 (<0.001) | 11.8122 (<0.001) | 11.0878 (<0.001) | 9.5584 (<0.001) |
| **CON04** | 14.5198 (<0.001) | 16.5587 (<0.001) | 13.5772 (<0.001) | 15.9567 (<0.001) | 15.9782 (<0.001) | 11.8693 (<0.001) | 13.5435 (<0.001) | 12.9962 (<0.001) | 12.3340 (<0.001) | 11.8500 (<0.001) | 10.8504 (<0.001) |
| **CON05** | 13.6438 (<0.001) | 14.1646 (<0.001) | 14.3904 (<0.001) | 13.7635 (<0.001) | 13.1815 (<0.001) | 15.2092 (<0.001) | 13.3642 (<0.001) | 12.6545 (<0.001) | 12.7487 (<0.001) | 10.7261 (<0.001) | 10.2549 (<0.001) |
| **CON06** | 14.2882 (<0.001) | 14.1018 (<0.001) | 14.0598 (<0.001) | 13.1669 (<0.001) | 12.8092 (<0.001) | 12.5303 (<0.001) | 12.5974 (<0.001) | 11.6257 (<0.001) | 12.6239 (<0.001) | 11.6692 (<0.001) | 12.2056 (<0.001) |
| **CON07** | 14.5607 (<0.001) | 15.8613 (<0.001) | 12.5815 (<0.001) | 14.6058 (<0.001) | 14.0954 (<0.001) | 14.0853 (<0.001) | 12.6214 (<0.001) | 12.0756 (<0.001) | 12.8695 (<0.001) | 13.8146 (<0.001) | 10.3861 (<0.001) |
| **CON08** | 14.8703 (<0.001) | 14.2441 (<0.001) | 13.1207 (<0.001) | 12.8119 (<0.001) | 11.5125 (<0.001) | 12.5454 (<0.001) | 13.0763 (<0.001) | 14.1522 (<0.001) | 12.4313 (<0.001) | 14.2165 (<0.001) | 12.1066 (<0.001) |
| **CON09** | 13.6107 (<0.001) | 15.1382 (<0.001) | 13.2348 (<0.001) | 14.9631 (<0.001) | 12.8602 (<0.001) | 12.7348 (<0.001) | 11.3155 (<0.001) | 13.4578 (<0.001) | 11.4187 (<0.001) | 13.7156 (<0.001) | 14.2129 (<0.001) |
| **CON10** | 13.9932 (<0.001) | 14.3101 (<0.001) | 12.1814 (<0.001) | 14.3921 (<0.001) | 13.1667 (<0.001) | 13.7562 (<0.001) | 13.3849 (<0.001) | 12.8326 (<0.001) | 12.1082 (<0.001) | 10.3231 (<0.001) | 12.7126 (<0.001) |
| **CON11** | 15.7275 (<0.001) | 13.2736 (<0.001) | 15.4348 (<0.001) | 14.7189 (<0.001) | 15.1149 (<0.001) | 15.0262 (<0.001) | 13.8375 (<0.001) | 14.5329 (<0.001) | 12.6317 (<0.001) | 13.1455 (<0.001) | 12.4558 (<0.001) |
| **CON12** | 13.4319 (<0.001) | 14.3731 (<0.001) | 13.1401 (<0.001) | 14.2131 (<0.001) | 12.4026 (<0.001) | 11.3156 (<0.001) | 12.8173 (<0.001) | 13.1923 (<0.001) | 12.2212 (<0.001) | 13.1763 (<0.001) | 14.1061 (<0.001) |
| **CON13** | 12.1263 (<0.001) | 11.4642 (<0.001) | 11.1212 (<0.001) | 13.8132 (<0.001) | 13.1156 (<0.001) | 11.3227 (<0.001) | 13.1218 (<0.001) | 11.5451 (<0.001) | 12.3029 (<0.001) | 12.2351 (<0.001) | 9.5986 (<0.001) |
| **CON14** | 13.2781 (<0.001) | 11.0312 (<0.001) | 12.8842 (<0.001) | 13.9601 (<0.001) | 13.1672 (<0.001) | 13.2941 (<0.001) | 14.3522 (<0.001) | 12.3184 (<0.001) | 13.6962 (<0.001) | 11.1253 (<0.001) | 12.1207 (<0.001) |
| **CON15** | 15.6671 (<0.001) | 14.1613 (<0.001) | 14.7217 (<0.001) | 12.7785 (<0.001) | 12.5218 (<0.001) | 10.1562 (<0.001) | 12.1417 (<0.001) | 11.9152 (<0.001) | 13.7128 (<0.001) | 11.7288 (<0.001) | 12.8532 (<0.001) |
| **CON16** | 12.7943 (<0.001) | 13.1274 (<0.001) | 13.2915 (<0.001) | 14.7064 (<0.001) | 13.8182 (<0.001) | 14.8346 (<0.001) | 13.3357 (<0.001) | 14.3874 (<0.001) | 12.9081 (<0.001) | 10.5097 (<0.001) | 12.0874 (<0.001) |
| **CON17** | 14.4228 (<0.001) | 12.6827 (<0.001) | 12.8152 (<0.001) | 14.1371 (<0.001) | 14.4201 (<0.001) | 13.2405 (<0.001) | 12.4843 (<0.001) | 12.6257 (<0.001) | 12.3963 (<0.001) | 11.9771 (<0.001) | 12.2153 (<0.001) |
| **CON18** | 13.6637 (<0.001) | 15.4663 (<0.001) | 13.0405 (<0.001) | 14.3259 (<0.001) | 12.8011 (<0.001) | 13.7631 (<0.001) | 12.2174 (<0.001) | 12.5722 (<0.001) | 10.1574 (<0.001) | 11.5424 (<0.001) | 10.2675 (<0.001) |
| **CON19** | 14.7184 (<0.001) | 13.3371 (<0.001) | 14.4112 (<0.001) | 13.7678 (<0.001) | 14.9445 (<0.001) | 13.2317 (<0.001) | 14.2323 (<0.001) | 11.4233 (<0.001) | 13.7286 (<0.001) | 12.1531 (<0.001) | 10.4339 (<0.001) |
| **CON20** | 12.1472 (<0.001) | 12.1295 (<0.001) | 13.2022 (<0.001) | 13.1283 (<0.001) | 12.6437 (<0.001) | 12.4761 (<0.001) | 13.2863 (<0.001) | 12.2569 (<0.001) | 12.2158 (<0.001) | 10.2561 (<0.001) | 11.5292 (<0.001) |

| | | | | | | | | | | | |
|---|---|---|---|---|---|---|---|---|---|---|---|
| **CON21** | 12.1042 (<0.001) | 13.0249 (<0.001) | 13.1497 (<0.001) | 11.3026 (<0.001) | 12.2921 (<0.001) | 11.8957 (<0.001) | 12.1283 (<0.001) | 11.8771 (<0.001) | 11.2572 (<0.001) | 11.2252 (<0.001) | 9.5186 (<0.001) |
| **CON22** | 13.1151 (<0.001) | 13.6529 (<0.001) | 11.3578 (<0.001) | 13.3612 (<0.001) | 13.7206 (<0.001) | 10.8596 (<0.001) | 13.1489 (<0.001) | 12.3487 (<0.001) | 11.8788 (<0.001) | 10.1354 (<0.001) | 12.6116 (<0.001) |
| **CON23** | 12.7361 (<0.001) | 14.2352 (<0.001) | 12.1211 (<0.001) | 14.1492 (<0.001) | 12.5861 (<0.001) | 13.9873 (<0.001) | 12.8612 (<0.001) | 12.3541 (<0.001) | 11.9439 (<0.001) | 12.1221 (<0.001) | 9.8293 (<0.001) |
| **CON24** | 11.6431 (<0.001) | 13.5348 (<0.001) | 13.1527 (<0.001) | 12.2235 (<0.001) | 13.4768 (<0.001) | 13.5104 (<0.001) | 14.7126 (<0.001) | 14.5669 (<0.001) | 10.6211 (<0.001) | 12.5343 (<0.001) | 11.5262 (<0.001) |

For the statistics based on thresholded graphs, comparisons were conducted over the range from 0.01 to 0.03 for each subject. All of the structural brain networks of CAE patients and healthy controls were significantly more modular than null models. CAE = childhood absence epilepsy, CON = healthy controls.

## Supplementary Table S3. The distribution of network parameters

The p-values of the null hypothesis(‘the data are normally distributed’) in patients.

| **Threshold** | **Strength** | **E***glob* | **E***loc* | **C**p | **L**p | **γ** | **λ** | **σ** | **E***nodal* **of PCUN_L** | **E***nodal* **of PCUN_R** | **Num_of_mod** | **Modularity** |
|---|---|---|---|---|---|---|---|---|---|---|---|---|
| 0.010 | 0.50[a] | 0.50[a] | 0.50[a] | 0.31 | 0.50[a] | 0.46 | 0.50[a] | 0.50[a] | 0.20 | 0.17 | 0.38 | 0.50[a] |
| 0.012 | 0.50[a] | 0.50[a] | 0.50[a] | 0.50[a] | 0.50[a] | 0.50[a] | 0.38 | 0.50[a] | 0.25 | 0.22 | 0.27 | 0.49 |
| 0.014 | 0.50a | 0.50[a] | 0.50[a] | 0.50[a] | 0.50[a] | 0.50[a] | 0.50[a] | 0.41 | 0.19 | 0.24 | 0.34 | 0.38 |
| 0.016 | 0.50[a] | 0.50[a] | 0.21 | 0.05[a] | 0.50[a] | 0.18 | 0.50[a] | 0.26 | 0.12 | 0.14 | 0.17 | 0.50[a] |
| 0.018 | 0.50[a] | 0.50[a] | 0.16 | 0.37 | 0.50[a] | 0.13 | 0.13 | 0.09 | 0.49 | 0.06 | 0.09 | 0.50[a] |
| 0.020 | 0.50[a] | 0.50a | 0.05 | 0.38 | 0.50[a] | 0.09 | 0.50[a] | 0.13 | 0.36 | 0.21 | 0.30 | 0.50[a] |
| 0.022 | 0.50[a] | 0.50[a] | 0.24 | 0.18 | 0.50[a] | 0.50[a] | 0.50[a] | 0.50[a] | 0.08 | 0.06 | 0.08 | 0.50[a] |
| 0.024 | 0.50[a] | 0.50[a] | 0.27 | 0.06 | 0.50[a] | 0.34 | 0.50[a] | 0.13 | 0.09 | 0.20 | 0.10 | 0.50[a] |
| 0.026 | 0.50[a] | 0.50[a] | 0.06 | 0.23 | 0.50[a] | 0.14 | 0.36 | 0.41 | 0.06 | 0.06 | 0.06 | 0.19 |
| 0.028 | 0.50[a] | 0.50[a] | 0.19 | 0.15 | 0.50[a] | 0.17 | 0.05[a] | 0.11 | 0.06 | 0.23 | 0.21 | 0.50[a] |
| 0.030 | 0.50[a] | 0.50[a] | 0.36 | 0.11 | 0.50[a] | 0.30 | 0.36 | 0.17 | 0.13 | 0.09 | 0.10 | 0.50[a] |
| integrated | 0.50[a] | 0.50[a] | 0.35 | 0.06 | 0.50[a] | 0.35 | 0.42 | 0.38 | 0.18 | 0.50[a] | - | - |

*a*: It returns 0.50 when P is greater than the largest tabulated value in ‘*lillietest*’. Num_of_mod = Number of modules.

The p-values of the null hypothesis(‘the data are normally distributed’) in controls.

| **Threshold** | **Strength** | **E***glob* | **E***loc* | **C**p | **L**p | **γ** | **λ** | **σ** | **E***nodal* **of PCUN_L** | **E***nodal* **of PCUN_R** | **Mum_of_mod** | **Modularity** |
|---|---|---|---|---|---|---|---|---|---|---|---|---|
| 0.010 | 0.07 | 0.12 | 0.32 | 0.50[a] | 0.10 | 0.33 | 0.09 | 0.18 | 0.06 | 0.15 | 0.13 | 0.34 |
| 0.012 | 0.06 | 0.22 | 0.07 | 0.50[a] | 0.17 | 0.35 | 0.09 | 0.33 | 0.15 | 0.07 | 0.46 | 0.50[a] |
| 0.014 | 0.12 | 0.12 | 0.21 | 0.50[a] | 0.10 | 0.42 | 0.07 | 0.21 | 0.12 | 0.16 | 0.50[a] | 0.21 |
| 0.016 | 0.32 | 0.17 | 0.10 | 0.39 | 0.12 | 0.36 | 0.44 | 0.15 | 0.09 | 0.17 | 0.27 | 0.27 |
| 0.018 | 0.15 | 0.12 | 0.09 | 0.50[a] | 0.10 | 0.12 | 0.06 | 0.11 | 0.21 | 0.19 | 0.11 | 0.50[a] |
| 0.020 | 0.29 | 0.12 | 0.28 | 0.32 | 0.11 | 0.18 | 0.35 | 0.18 | 0.15 | 0.11 | 0.14 | 0.50[a] |
| 0.022 | 0.23 | 0.11 | 0.13 | 0.31 | 0.09 | 0.50[a] | 0.31 | 0.50[a] | 0.14 | 0.20 | 0.11 | 0.50[a] |
| 0.024 | 0.09 | 0.07 | 0.50[a] | 0.40 | 0.07 | 0.50[a] | 0.48 | 0.28 | 0.23 | 0.08 | 0.27 | 0.50[a] |
| 0.026 | 0.09 | 0.09 | 0.11 | 0.34 | 0.08 | 0.21 | 0.23 | 0.05 | 0.06 | 0.13 | 0.08 | 0.50[a] |
| 0.028 | 0.18 | 0.29 | 0.28 | 0.50[a] | 0.25 | 0.50[a] | 0.50[a] | 0.50[a] | 0.23 | 0.13 | 0.27 | 0.25 |
| 0.030 | 0.09 | 0.20 | 0.10 | 0.41 | 0.17 | 0.50[a] | 0.13 | 0.39 | 0.08 | 0.19 | 0.11 | 0.50[a] |
| integrated | 0.08 | 0.12 | 0.49 | 0.10 | 0.11 | 0.50[a] | 0.50[a] | 0.15 | 0.10 | 0.23 | - | - |

*a*: It returns 0.50 when P is greater than the largest tabulated value in ‘*lillietest*’. Num_of_mod = Number of modules.
